# *GNAS* AS2 methylation status enables mechanism-based categorization of pseudohypoparathyroidism type 1B

**DOI:** 10.1172/jci.insight.177190

**Published:** 2024-01-30

**Authors:** Yorihiro Iwasaki, Monica Reyes, Harald Jüppner, Murat Bastepe

**Affiliations:** 1 Endocrine Unit, Department of Medicine, Massachusetts General Hospital and Harvard Medical School, Boston, Massachusetts, USA.; 2 Tazuke Kofukai Medical Research Institute, Kitano Hospital, Osaka, Japan.; 3 Pediatric Nephrology Unit, Massachusetts General Hospital and Harvard Medical School, Boston, Massachusetts, USA.

**Keywords:** Endocrinology, Genetics, G proteins, Imprinting, Molecular genetics

## Abstract

Pseudohypoparathyroidism type 1B (PHP1B) results from aberrant genomic imprinting at the *GNAS* gene. Defining the underlying genetic cause in new patients is challenging because various genetic alterations (e.g., deletions, insertions) within the *GNAS* genomic region, including the neighboring *STX16* gene, can cause PHP1B, and the genotype-epigenotype correlation has not been clearly established. Here, by analyzing patients with PHP1B with a wide variety of genotypes and epigenotypes, we identified a *GNAS* differentially methylated region (DMR) of distinct diagnostic value. This region, *GNAS* AS2, was hypomethylated in patients with genetic alterations located centromeric but not telomeric of this DMR. The AS2 methylation status was captured by a single probe of the methylation-sensitive multiplex ligation–dependent probe amplification (MS-MLPA) assay utilized to diagnose PHP1B. In human embryonic stem cells, where NESP55 transcription regulates *GNAS* methylation status on the maternal allele, AS2 methylation depended on 2 imprinting control regions (STX16-ICR and NESP-ICR) essential for NESP55 transcription. These results suggest that the AS2 methylation status in patients with PHP1B reflects the position at which the genetic alteration affects NESP55 transcription during an early embryonic period. Therefore, AS2 methylation levels can enable mechanistic PHP1B categorization based on genotype-epigenotype correlation and, thus, help identify the underlying molecular defect in patients.

## Abstract 2

A2 methylation of <em>GNAS</em> is regulated by STX16 and NESP55 imprinting control regions, and <em>GNAS </em>A2 methylation status enables pseudohypoparathyroidism type 1B categorization.

## Graphical abstract

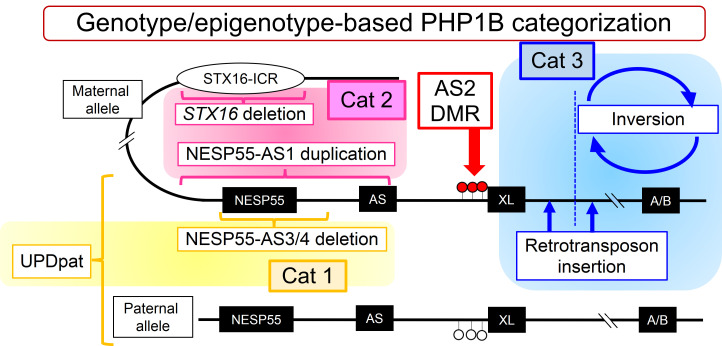

## Introduction

The *GNAS* gene locus (chromosome 20q13.32) encodes the α-subunit of stimulatory G protein (Gsα) that mediates signaling of a broad range of G protein–coupled receptors. Genetic abnormalities affecting *GNAS* are involved in the pathogenesis of various diseases, including endocrine disorders and cancers (1–5). Pseudohypoparathyroidism (PHP) is one of those disorders characterized by resistance to parathyroid hormone (PTH) and several other hormones that is caused by genetic alterations that impair the expression or function of Gsα (5). Since its first description as the mammalian prototype of end-organ hormone resistance (6), 2 major subtypes and 1 related disorder have been linked to distinct types of genetic/epigenetic alterations affecting *GNAS*: (a) PHP type 1A (PHP1A), caused by inactivating Gsα mutations on the maternal allele; (b) PHP1B, caused by *GNAS* epigenetic defects; and (c) pseudo-PHP (PPHP) caused by inactivating Gsα mutations on the paternal allele. While patients with PHP1A show PTH resistance with physical findings known as Albright’s hereditary osteodystrophy (AHO) (short stature, brachydactyly, round face, obesity, and other features) (4, 6), patients with PPHP lack hormone resistance but display AHO. In contrast, patients with PHP1B typically present with PTH and, often, TSH resistance without most AHO features and only rarely with obvious AHO.

**Figure 1:**
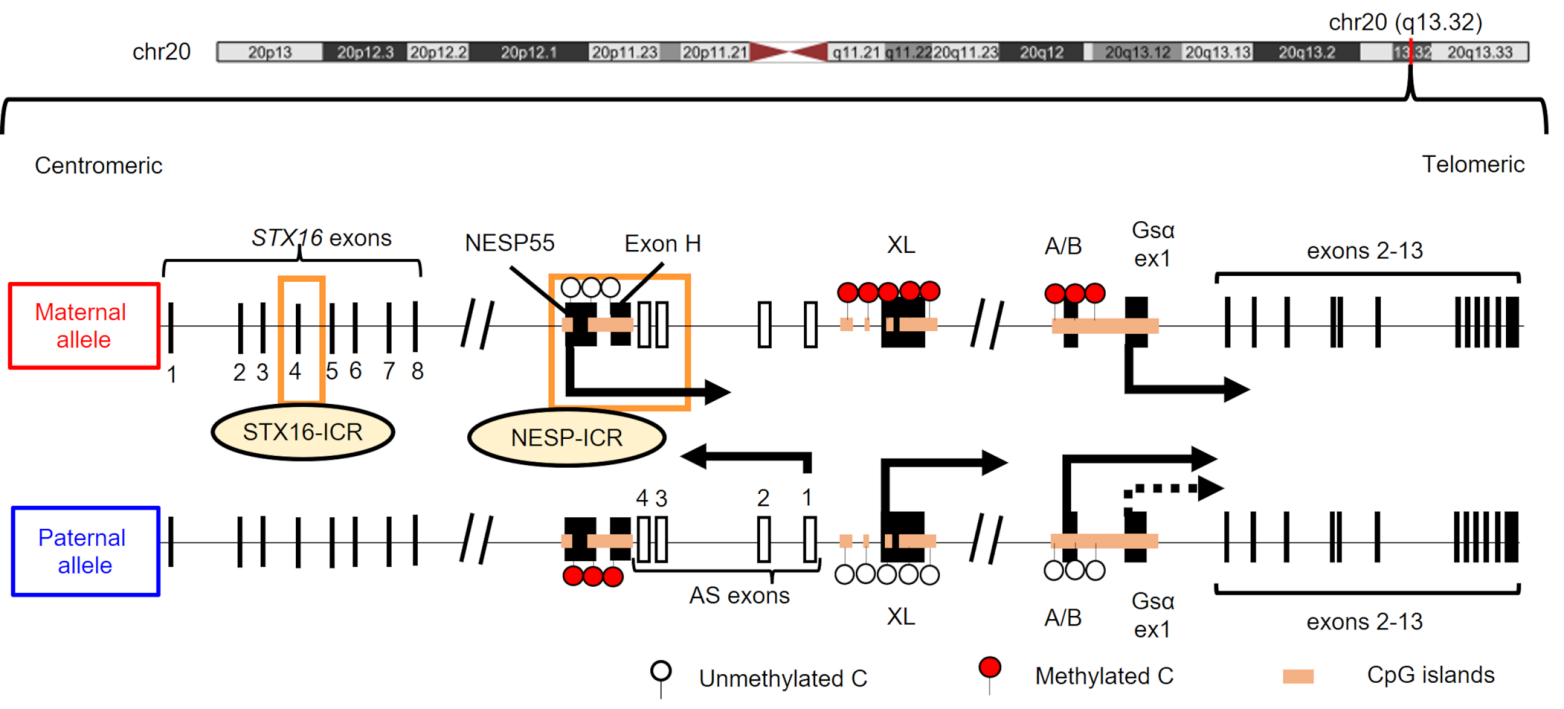
Schematic representation of the *GNAS* gene locus. Red and white circles depict methylated and unmethylated DMRs, respectively. Orange boxes indicate imprinting control regions (ICRs) in the *STX16* locus (STX16-ICR) or the region surrounding the NESP55 exon within the *GNAS* locus (NESP-ICR). Arrows indicate the transcriptional direction. A dashed arrow from paternal Gsα exon 1 (ex1) indicates silenced Gsα transcription.

The complex phenotypes of different PHP variants are attributable to genomic imprinting of several *GNAS* exons and the finding that multiple transcripts are derived from this locus. In addition to Gsα-encoding exon 1–13, the *GNAS* locus comprises several alternative first exons centromeric (upstream) of exon 1, including NESP55, XL, and A/B (Figure 1). An additional antisense (AS) transcript originates from a region centromeric (upstream) of XL. Each alternative first exon, as well as the first exon of the AS transcript, is differentially methylated in a parental allele-specific manner. The NESP55 differentially methylated region (DMR) is paternally methylated and is maternally transcribed. The AS, XL, and A/B DMRs have maternal methylation and are paternally transcribed. Although the first exon of Gsα does not have a DMR, transcription from its paternal allele is silenced through an unknown mechanism in specific tissues, such as proximal renal tubules and thyroid gland (7, 8).

In PHP1B, all patients show severely reduced methylation at the A/B DMR, accompanied, in some cases, by methylation alterations at remaining *GNAS* DMRs. Distinct genetic alterations identified in some patients with PHP1B have substantially contributed to understanding the regulatory mechanism of *GNAS* imprinting. Following the initial discovery of a recurrent chromosomal microdeletion affecting the *STX16* gene locus in autosomal dominant–PHP1B (AD-PHP1B) kindreds (9), various genetic alterations have been identified in other patients with AD-PHP1B: chromosomal deletions involving the NESP55 exon and/or AS exons 3/4, chromosomal duplications, a chromosomal inversion, and retrotransposon insertions (5, 10–15). Since these genetic abnormalities cause PHP solely when located on the maternal allele, they likely disrupt the regulatory mechanism of *GNAS* imprinting. The underlying defect is unknown in the majority of sporadic PHP1B cases, although several patients were found to have paternal uniparental disomy (UPDpat) involving the chromosomal region comprising *GNAS* (4, 16). Despite its importance in PHP1B diagnosis, identifying the genetic cause is challenging because of the wide range of possibilities that are not systematically categorized (5).

Patients with PHP1B show specific *GNAS* methylation patterns corresponding to underlying genetic alterations (4, 5). For instance, *STX16* deletions — the most common cause of AD-PHP1B — lead to loss of methylation at A/B, with normal methylation levels at NESP55, AS, and XL (5, 9). In contrast, deletion of AS exons 3/4 causes broad methylation defects, including incomplete gain of methylation at NESP55 and loss of methylation at all remaining maternal DMRs (5, 10, 17). While such genotype-epigenotype correlation is informative for molecular diagnosis of PHP1B, the mechanistic basis of this correlation has been poorly understood.

Until recently, the *GNAS* methylation pattern in patients with deletions affecting *STX16* was thought to be limited to isolated A/B hypomethylation. Mechanistically, *STX16* was shown to operate as an early embryonic stage–specific enhancer for NESP55 transcription, which is essential for methylating the maternal A/B DMR (18). However, a recent study identified an additional DMR between AS exon 1 and the XL exon, referred to as AS2, that shows hypomethylation in patients with PHP1B with *STX16* deletions (19). While the role of the AS2 DMR in regulating *GNAS* transcripts has not yet been addressed, the methylation of this region is not reduced in more recently described patients with PHP1B with isolated A/B hypomethylation who carry retrotransposon insertions within *GNAS* as the associated genetic alteration (12, 20). These findings suggest that the methylation status of the AS2 DMR contributes to the complex genotype-epigenotype correlation in PHP1B and may, therefore, help categorize the patients according to underlying genetic causes.

In the current study, we took advantage of our broad range of clinical samples obtained from rare patients with PHP1B and determined that AS2 methylation is differentially affected based on the genetic alteration responsible for the disease. We also identified a probe that reflects AS2 methylation within the methylation-sensitive multiplex ligation-dependent probe amplification (MS-MLPA) assay utilized commonly to characterize the epigenetic and genetic features of patients with PHP1B. Moreover, using our recently developed hESC-based PHP1B models (18), we found that AS2 methylation is regulated in the early embryo by the *GNAS* imprinting control regions (ICRs) regulating NESP55 transcription and exon A/B methylation. Our study indicates that the status of AS2 methylation is valuable for the genotype-epigenotype correlation of PHP1B cases, thus guiding efforts to identify the underlying genetic cause. Our work provides the first mechanism-based PHP1B categorization, to our knowledge, immediately applicable to molecular diagnosis.

## Results

### Methylation levels upstream of the XL exon are differentially affected according to underlying defects in patients with PHP1B.

**Figure 2:**
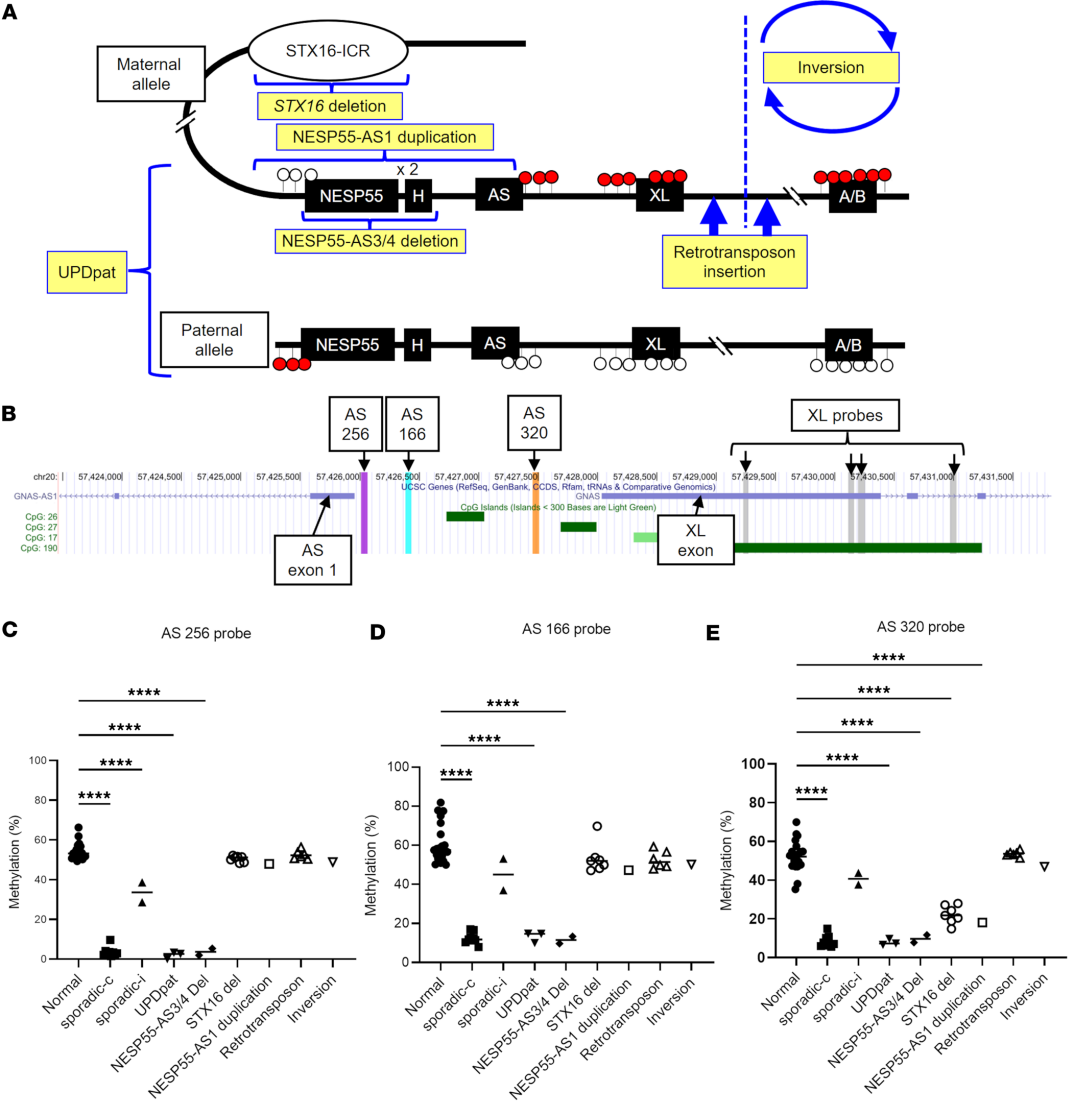
Differential effects of PHP1B genetic alterations on MS-MLPA probes. (**A**) Schematic representations of underlying genetic alterations in PHP1B patient samples studied. Red and white circles depict methylated and unmethylated DMRs, respectively. (**B**) A UCSC genome browser track showing the chromosomal locations of MS-MLPA probes in the AS-XL region. The 256, the 166, and the 320 probes are the 3 probes designed in the AS DMR region. (**C**–**E**) MS-MLPA results of patients with PHP1B (*n* = 31) and unaffected controls (*n* = 21). Methylation levels at the 256 probe (**C**), the 166 probe (**D**), and the 320 probe (**E**) are shown. In **E**, values in the *STX16* deletion group were significantly higher than in the sporadic-c group (*P* = 0.0003). Intergroup comparisons were performed by 1-way ANOVA with post hoc Dunnett multiple comparison test. ****P < 0.0001. Sporadic-c, sporadic cases with complete methylation defects; Sporadic-i, sporadic cases with incomplete methylation defects; UPDpat, paternal uniparental disomy of chromosomal 20; NESP55-AS3/4 del, maternal deletion of NESP55-AS exons 3/4 region; STX16 del, maternal *STX16* deletion; NESP55-AS1 duplication, duplication comprising the maternal NESP55-AS exon 1 (excluding the region between the AS exon 1 and the XL exon). Retrotransposon insertion idicates retrotransposon insertion telomeric (downstream) of the maternal XL exon. Inversion indicates maternal inversion involving A/B and all Gsα exons with a centromeric (upstream) breakpoint between XL and A/B.

To analyze the *GNAS* methylation status of patients with PHP1B (*n* = 31 in total) with a variety of defined genetic alterations (*n* = 20 in total) (Figure 2A), genetically undefined sporadic cases (*n* = 11), and unaffected controls (*n* = 21), we first utilized a commercially available MS-MLPA kit, which has 3 probes in the region between AS exon 1 and the XL exon, hereafter referred to as AS 256, 166, and 320 probes based on the amplicon length (Figure 2B and Supplemental Table 1; supplemental material available online with this article; https://doi.org/10.1172/jci.insight.177190DS1). Consistent with previous findings (4, 5), methylation levels at the A/B DMR were significantly lower in all PHP1B cases than in unaffected controls (Supplemental Figure 1). Among patients with PHP1B, sporadic cases with undefined defects, UPDpat, and patients with a deletion involving NESP55-AS exons 3/4 region showed broad methylation defects, including hypomethylation at AS and XL (Supplemental Table 1), consistent with previous findings (4, 5, 10, 16). The remaining patients with PHP1B showed normal methylation levels at all MS-MLPA probes, except for 1 probe, the “320 probe,” which showed varying degrees of methylation (Figure 2, B–E, and Supplemental Table 1). The 320 probe is located in the region between AS and XL DMRs, slightly centromeric (upstream) of the XL exon (Figure 2B). Among patients with PHP1B who did not show *GNAS* broad methylation defects, the 320 probe methylation levels were significantly lower than normal in patients with maternal *STX16* deletion and a chromosomal duplication comprising the maternal NESP55-AS exon 1, which does not involve the region between the AS exon 1 and the XL exon (Figure 2E). On the other hand, the methylation levels did not differ significantly from normal in patients with PHP1B with 2 other defined genetic causes, a retrotransposon insertion telomeric (downstream) of the XL exon and a chromosomal inversion involving A/B and all Gsα exons with a centromeric breakpoint close to the retrotransposon insertion (but not involving the XL exon) (Figure 2E). These results suggest that, on top of conventionally analyzed *GNAS* methylation patterns, methylation levels at the 320 probe could have diagnostic potential for narrowing the location of the PHP1B-causing genetic mutation.

### The MS-MLPA probe of diagnostic potential reflects AS2 methylation levels.

**Figure 3:**
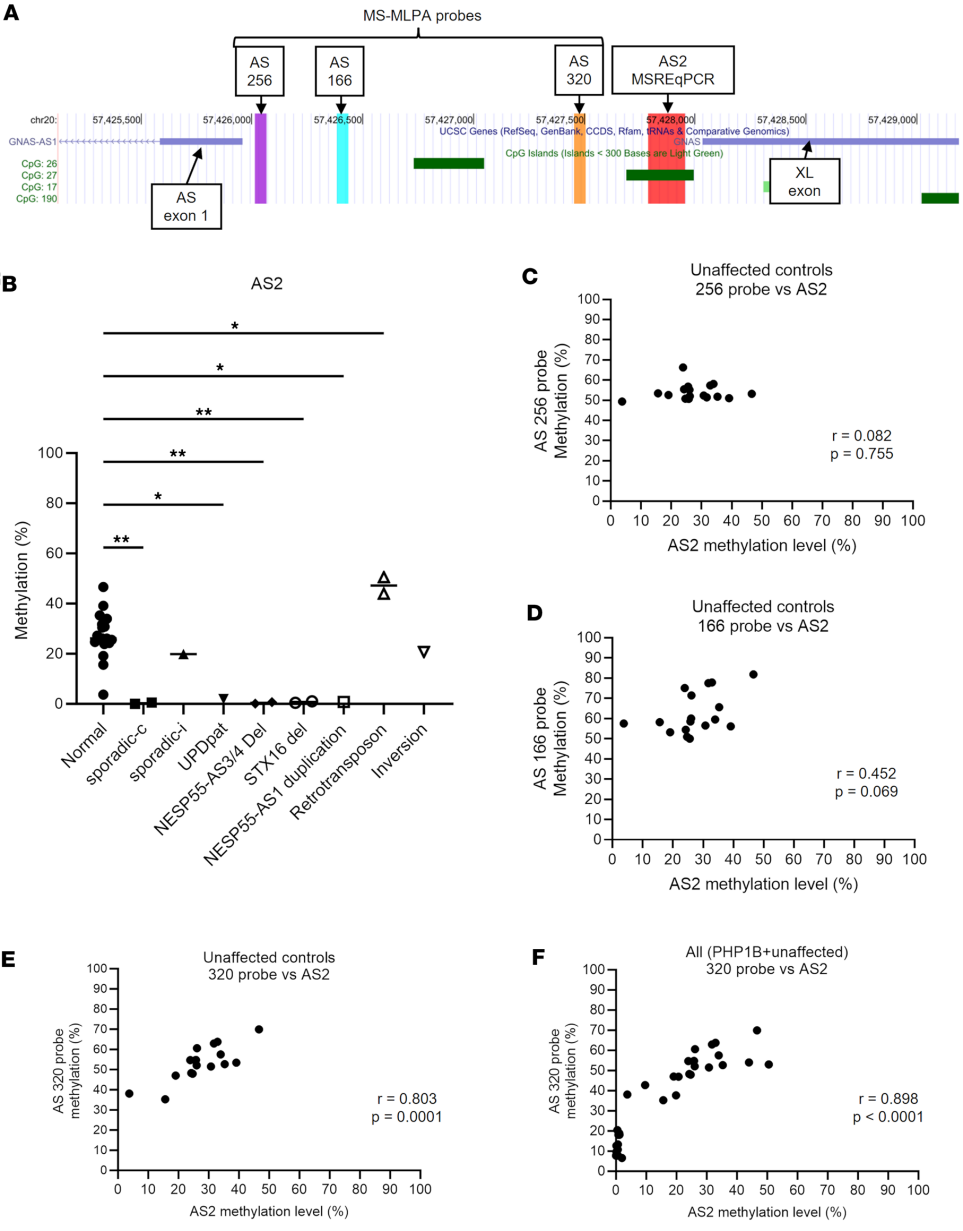
Diagnostic relevance of AS2 methylation levels and their correlation with the methylation levels at MS-MLPA probes in the AS region (**A**) A UCSC genome browser track showing the chromosomal locations of AS2 MSRE-qPCR amplicons, MS-MLPA probes, XL exon, and AS exon 1. (**B**) AS2 methylation levels measured by MSRE-qPCR in unaffected controls (*n* = 19) and various patients with PHP1B (*n* = 12). Intergroup comparisons were performed by 1-way ANOVA with post hoc Dunnett multiple comparison test. *P < 0.05, **P < 0.01. (**C**–**F**) Correlation between AS2 methylation levels measured by MSRE-qPCR and those at 3 MS-MLPA probes. Correlation of methylation levels between AS2 and the 256 (**C**), the 166 (**D**), and the 320 (**E**) probes in unaffected controls (*n* = 17). Correlation of methylation levels between AS2 and the 320 probes in all available samples (*n* = 29), including patients with PHP1B and unaffected controls (**F**). The Pearson correlation coefficients and the p values are shown.

Since the 320 probe is located ~200 bp centromeric (upstream) of the AS2 DMR (Figure 3A), we measured AS2 methylation levels using methylation-sensitive restriction enzyme quantitative PCR (MSRE-qPCR) in a subset of the cohort analyzed by MS-MLPA (19 unaffected controls and 12 patients with PHP1B with a variety of underlying genetic alterations or undetermined causes; Supplemental Table 1). The methylation level at the AS2 DMR in unaffected controls was highly variable (mean ± SEM, 27.5% ± 2.1%) but was, on average, lower than the expected 50% (Figure 3B). AS2 methylation was almost completely lost in patients with PHP1B who showed broad *GNAS* methylation defects (Supplemental Table 1) and in those with a maternal *STX16* deletion or a chromosomal duplication comprising NESP55-AS exon 1 (Figure 3B and Supplemental Table 1). On the other hand, AS2 methylation levels were close to normal levels in patients with PHP1B with a retrotransposon insertion telomeric (downstream) of the XL exon or a chromosomal inversion involving A/B and all Gsα exons (Figure 3B and Supplemental Table 1). Based on the similarity of methylation patterns at the 320 probe and the AS2 DMR, we compared methylation levels at the AS2 DMR and the 320 probe. AS2 methylation levels were significantly correlated with methylation levels at the 320 probe but not with other nearby probes in unaffected control (Figure 3, C–E). A similar correlation between AS2 methylation and the 320 probe was also observed in all (unaffected + PHP1B) samples (Figure 3F). These results indicate that methylation levels at the 320 probe and the AS2 DMR are equivalently regulated and are affected only by specific genetic causes underlying PHP1B.

### AS2 methylation depends on both STX16- and NESP-ICRs.

**Figure 4:**
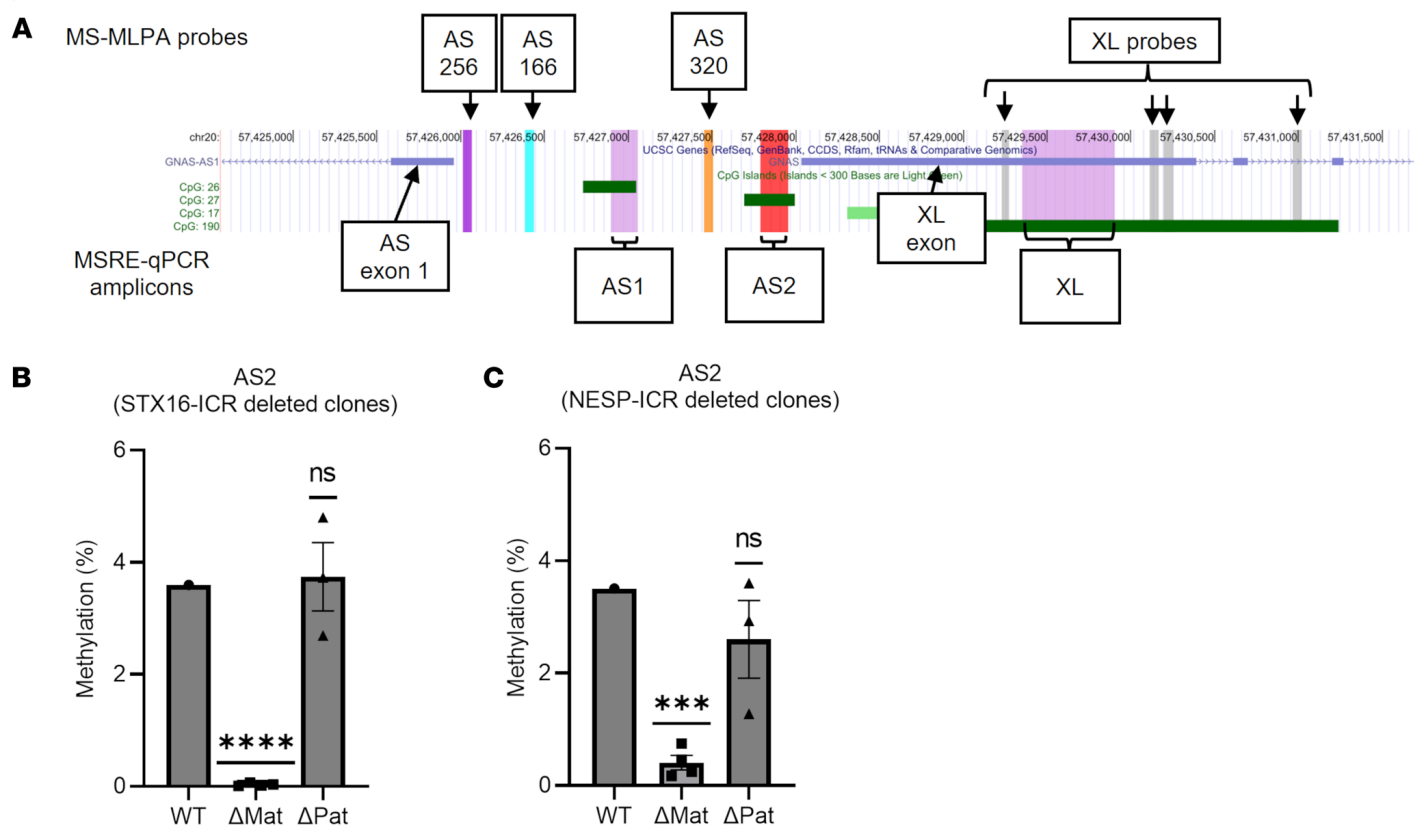
AS2 methylation levels in hESCs with *GNAS* ICR deletions. (**A**) A UCSC genome browser track showing the chromosomal locations of MSRE-qPCR amplicons (AS1, AS2, and XL) used for methylation analysis of hESCs. The AS2 DMR was analyzed in this study, and surrounding DMRs, AS1 and XL, were analyzed in our previous study (18). Locations of MS-MLPA probes and XL exon and AS exon 1 are also shown. (**B** and **C**) Methylation levels at the AS2 DMR in hESCs analyzed by MSRE-qPCR. Each dot represents an independent hESC clone. Results in wild-type (WT) hESCs and STX16-ICR maternally (ΔMat, *n* = 4) or paternally (ΔPat, *n* = 3) deleted hESC clones are shown (**B**). Results in WT hESCs and NESP-ICR maternally (ΔMat, *n* = 4) or paternally (ΔPat, *n* = 3) deleted hESC clones are shown (**C**). WT vs ΔPat or ΔMat values were compared using the one-sample t-tests. ***P < 0.001, ****P < 0.0001.

Based on the result that AS2 methylation levels are almost entirely lost in patients with PHP1B with deletions in either the *STX16* or NESP55-AS exons 3/4 regions (Figure 3B), we hypothesized that these regions are involved in a previously uncharacterized mechanism regulating AS2 methylation. Two ICRs, NESP-ICR and STX16-ICR, are required for A/B methylation on the maternal allele in an early embryonic period and possibly during oogenesis (18, 21). Accordingly, we used hESCs with either STX16-ICR or NESP-ICR deletion (18) to test which ICR affects AS2 methylation levels. WT hESCs showed lower AS2 methylation levels (~3.6%) by MSRE-qPCR compared with those observed in the leukocyte DNA from unaffected controls (3.73%–46.7%) (Figure 3B and Figure 4, A–C). Remarkably, AS2 methylation levels were significantly lower in the absence of either the STX16- or the NESP-ICR, specifically on the maternal allele but not on the paternal allele (Figure 4, B and C). The methylation levels at the flanking AS1 and XL DMRs were not reduced, as we have shown previously (18). These findings demonstrate that STX16- and NESP-ICRs are indispensable for AS2 methylation in an early embryonic period and, furthermore, suggest that *STX16* enhancer–driven NESP55 transcription specifically affects the AS2 DMR within the AS-XL region on the maternal allele.

### Retrotransposon insertion attenuates transcription.

**Figure 5:**
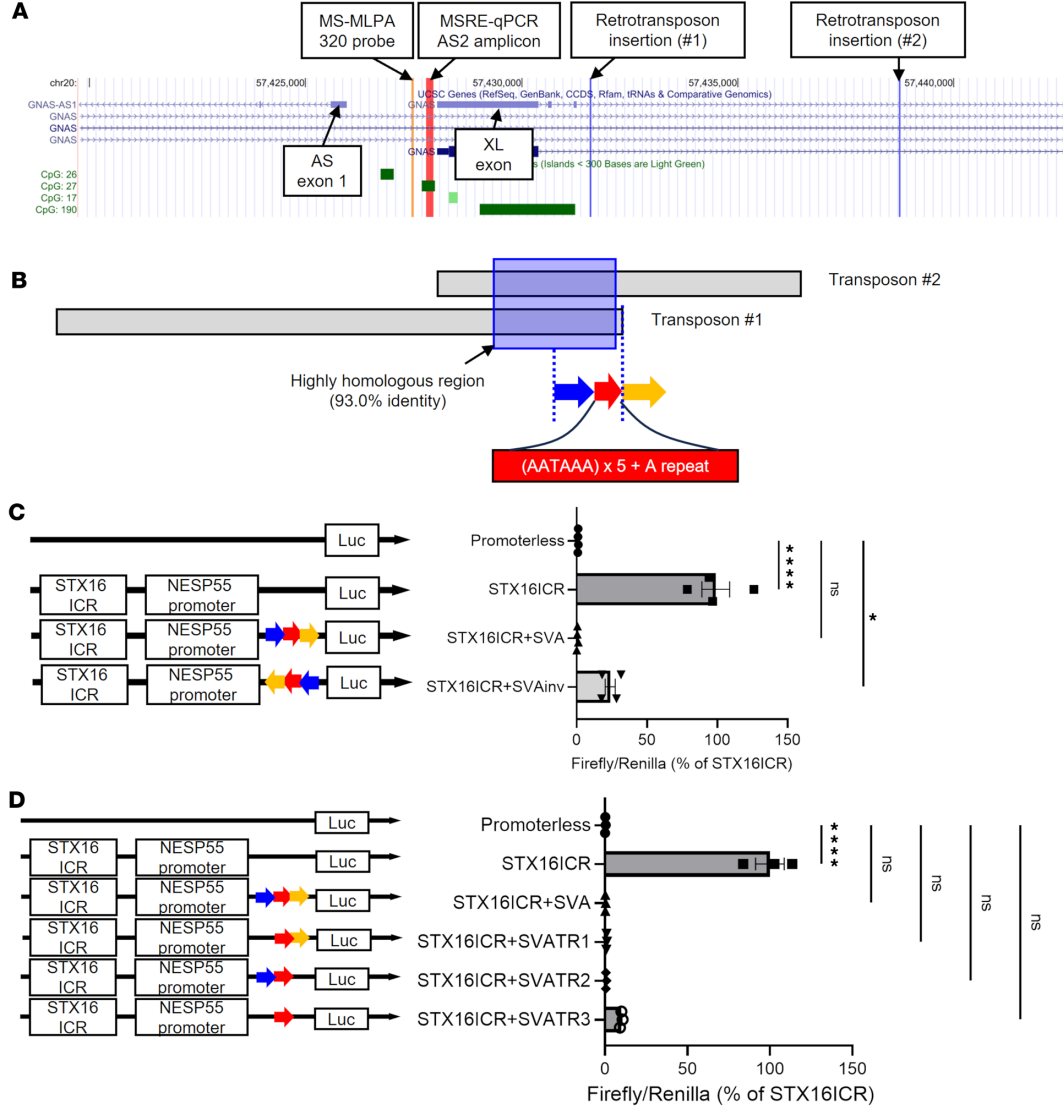
The effect of retrotransposon sequences on the passing-through transcription (**A**) A UCSC genome browser track showing the locations of retrotransposon insertions identified in 2 kindreds (#1 and #2; refs. 12, 14). Locations of the AS2 MSRE-qPCR amplicon and the 320 probe are also shown. (**B**) A schematic representation of the location of a highly homologous sequence in the retrotransposon identified in kindreds #1 and #2. The red arrow indicates the location of the tandemly repeated polyadenylation signal. Blue and yellow arrows indicate surrounding cloned regions for the reporter assay. (**C** and **D**) Luciferase assays in hESCs. Forty-eight hours following the transfection of each reporter plasmid in WT hESCs, firefly counts were measured and normalized using Renilla counts. The polyadenylation signal portion with surrounding sequences derived from kindred #1 was cloned into the STX16-ICR/NESP55 promoter-driven firefly luciferase plasmid (*n* = 4). Rightward and leftward arrows indicate sense and antisense orientation, respectively. STX16-ICR, NESP55 promoter and STX16-ICR; STX16-ICR+SVA, NESP55 promoter and STX16-ICR with sense-oriented insertion of transposon sequence; STX16-ICR+SVAinv, NESP55 promoter and STX16-ICR with antisense-oriented insertion of transposon sequence (**C**). Inserted kindred #1–derived sequence used in **C** was truncated as indicated (TR1-TR3) (*n* = 3) (**D**). Intergroup comparisons were performed by 1-way ANOVA with post hoc Dunnett multiple comparison test. *P < 0.05, ****P < 0.0001.

AS2 methylation was not reduced in patients with PHP1B with a retrotransposon insertion or a chromosomal inversion, as opposed to those with *STX16* deletions (Figure 2E and Figure 3B). Given that both the retrotransposon and the chromosomal inversion involve the maternal *GNAS* region downstream (telomeric) of the AS2 DMR, we hypothesized that AS2 methylation, unlike A/B methylation, was preserved in these cases because the NESP55 transcript was truncated between AS2 and A/B. To test this hypothesis, we focused on 1 of our PHP1B kindreds (family number 208, including 6 patients in Supplemental Table 1) with a retrotransposon insertion between AS2 and A/B, which is 1 of 2 such familial cases described to date (12, 14) (Figure 5A). Affected patients in both kindreds showed equivalent methylation patterns — i.e., A/B DMR was variably hypomethylated, but AS2 DMR was not hypomethylated (Figure 5A) (12, 14). The inserted sequences of both kindreds share substantial homology (~93% identity) over a ~600 bp region, followed by several tandem consensus polyadenylation signals at the telomeric end (Figure 5B and Supplemental Figure 2). To examine if these sequences might prematurely truncate the NESP55 transcript, we cloned the polyadenylation signals and the flanking sequences (from kindred #1 in Figure 5A) into luciferase reporter plasmids driven by the NESP55 promoter with *STX16* enhancer (18) and tested its effect in hESCs. Remarkably, including these patient-derived sequences suppressed the luciferase activity driven by the NESP55 promoter. This effect was significantly more profound when inserted in the same orientation as present in the patients’ genomes compared with the inverted orientation (Figure 5C). Furthermore, we generated various reporter constructs with truncated insertions to identify the critical portion that blunts transcription (Figure 5D). The middle segment alone, including the tandemly repeated polyadenylation signal, nearly abrogated the *STX16* enhancer/NESP55 promoter reporter activity. These results collectively support our hypothesis that the presence of the homologous portion in the inserted retrotransposon efficiently suppresses read-through transcription. Although the tandem polyadenylation signals in the retrotransposon likely play a critical role in impeding NESP55 transcription, additional surrounding sequences, especially the centromeric (upstream) portion, may also contribute to this effect.

### Mechanistic categorization of patients with PHP1B based on GNAS methylation defect patterns.

**Figure 6:**
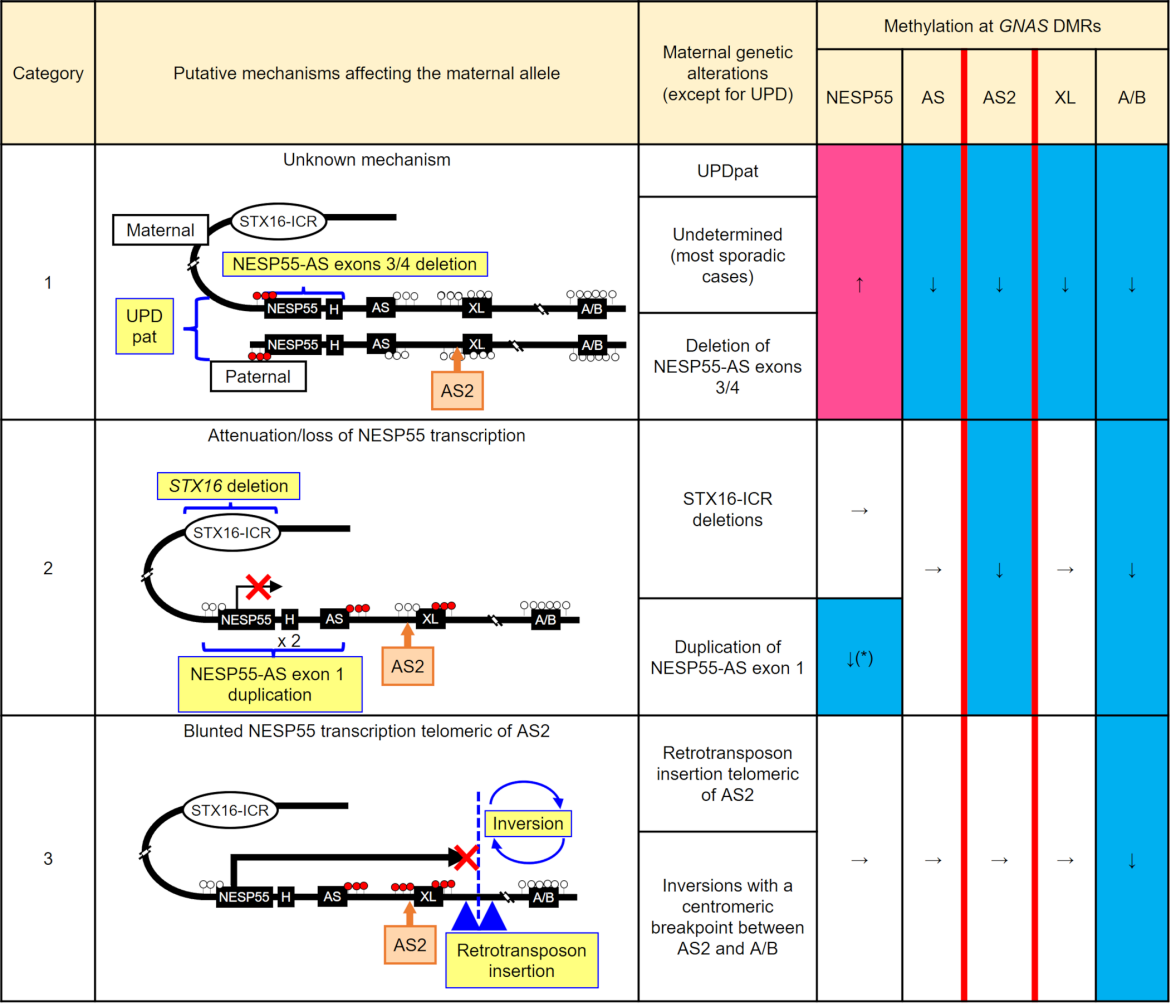
PHP1B categories based on pathogenic mechanisms and corresponding *GNAS* methylation patterns. Patients with PHP1B are classified into 3 categories based on *GNAS* methylation patterns. Category 1 is characterized by broad methylation defects caused by undetermined underlying causes (sporadic) with the exception of paternal uniparental disomy of chromosome 20 (UPDpat) and deletions comprising the NESP55-AS exons 3/4 region. Category 2 cases show a loss of methylation at AS2 and A/B while AS and XL methylation levels are preserved. Transcriptional attenuation of NESP55 centromeric (upstream) of the AS2 DMR causes this pattern, in which maternal *STX16* deletions are the most frequent cause. Category 3 is characterized by isolated A/B loss of methylation with preserved AS2 methylation levels, suggesting that NESP55 transcription is blunted telomeric (downstream) of the AS2 DMR. Asterisk indicates apparent hypomethylation due to copy number gain.

Based on the current findings, methylation levels at the AS2 DMR appear to reflect the locations of *GNAS* *cis*-regulatory mutations in PHP1B. Normal AS2 methylation is likely to be preserved only when read-through transcription from the NESP55 promoter is intact. Therefore, by combining AS2 methylation levels, which can be inferred from those measured by the MS-MLPA 320 probe, with those at the conventionally analyzed DMRs, patients with PHP1B can be categorized to reflect the location of *cis*-regulatory defects affecting the *GNAS* locus (Figure 6). Category 1 represents patients with broad methylation defects — i.e., a loss of methylation at all maternally methylated DMRs, including AS2, and a gain of methylation at the NESP55 DMR. Sporadic patients with PHP1B and those with maternal AS exon 3/4 deletion belong to this category. Category 2 comprises patients with a loss of methylation restricted to the A/B and the AS2 DMRs that is caused by impaired NESP55 transcription centromeric (upstream) of the AS2 DMR. This group includes patients with maternal *STX16* deletions and those with a maternal duplication of a region that extends from upstream of the NESP55 exon to downstream of AS exon 1 (excluding the AS2 DMR). Although we had no DNA samples from patients with deletions restricted to maternal NESP55 exon, their *GNAS* methylation pattern was reported to be similar to that of patients with *STX16* deletions (15, 22), suggesting that these patients should fall into category 2. Category 3 patients have bona fide isolated A/B hypomethylation with normal methylation status at the remaining DMRs, including the AS2 DMR. NESP55 transcription through the AS2 DMR should be intact in those cases. Therefore, this category includes maternal retrotransposon insertions telomeric (downstream) of the AS2 DMR and maternal chromosomal inversions with the centromeric breakpoint between XL and A/B.

## Discussion

This study identified *GNAS* AS2 methylation as a critical epigenetic alteration of PHP1B that can guide the search for the underlying disease-causing genetic alterations at *GNAS*/*STX16* regions. Our findings extend the current knowledge of *GNAS* epigenetic changes and allow several conclusions: (a) AS2 methylation levels enable mechanistic categorization of patients with PHP1B; (b) the 320 probe of the MS-MLPA assay reflects AS2 methylation levels and, thus, has diagnostic potential of clinical importance; and (c) AS2 methylation levels depend on *STX16* enhancer-driven NESP55 transcription, most likely at an early embryonic stage.

Molecular diagnosis of PHP1B primarily depends on *GNAS* methylation analysis, for which MS-MLPA is the most widely applied method (4). Even though several genetic and epigenetic abnormalities have been described in patients with PHP1B, the search for genetic alterations on the basis of the known mechanism resulting in epigenetic changes has thus far not been available. We showed that a single MS-MLPA probe, the 320 probe, revealed varying degrees of methylation in patients with PHP1B with different genetic alterations and that the methylation level at the 320 probe significantly correlated with the methylation level at the nearby AS2 DMR. Since AS2 methylation levels enable mechanism-based categorization of PHP1B, as discussed below, our characterization of the 320 probe has an important clinical implication.

This study also provides the first experimental evidence, to our knowledge, showing that STX16- and NESP-ICRs are indispensable for AS2 methylation at an early embryonic stage. Because AS2 is located just centromeric (upstream) of the XL exon, this region may operate as a promoter for XLαs transcription. Although further experimental validation is needed, lower AS2 methylation levels in hESCs could explain the biallelic XLαs expression previously observed for these cells (18). Notably, we found in this study that AS2 methylation levels in hESCs were almost completely lost upon deletion of either the maternal STX16-ICR or the maternal NESP-ICR, indicating dependency of the AS2 DMR on these *GNAS* ICRs located further centromeric. This is similar to the A/B DMR, for which a nascent NESP55 transcript driven by the *STX16* enhancer is necessary for its methylation (18). It is possible that A/B methylation on the maternal *GNAS* allele is established during oogenesis (21), similar to other maternally methylated imprinted loci (23). However, in a postzygotic period, A/B undergoes a second wave of demethylation and remethylation, which is unusual for a germline imprinted locus (24, 25). Mechanistically, A/B methylation shows higher sensitivity to inhibition of maintenance DNA methylase, DNMT1, in comparison with other representative maternally imprinted loci in hESCs, and it appears that *STX16* enhancer–driven NESP55 transcription is required to remethylate A/B after fertilization (18). Likewise, our current findings in hESCs indicate that the *STX16* enhancer–driven NESP55 transcription critically regulates AS2 methylation during the postzygotic period. On the other hand, methylation dynamics at AS2 during oogenesis, in the zygote, and at different stages of postzygotic development remains to be determined. A mouse study shows that the DMR spanning the region that extends from the AS exon 1 to the XL exon, which includes the AS2 DMR in humans, contains a female germline imprint mark (26). Since hESC models do not recapitulate epigenetic reprogramming during gametogenesis, we cannot rule out the possibility that STX16- and NESP-ICRs also play a role in the establishment of AS2 methylation during oogenesis.

Analysis of patients with PHP1B with various underlying defects provided results that are consistent with our hESC findings. Among patients with PHP1B without broad methylation alterations, AS2 methylation levels were decreased selectively, albeit to variable degrees, in patients with genetic causes that blunt NESP55 transcription upstream (centromeric) of the AS2 DMR. These include the following defects: maternal *STX16* deletion and maternal chromosomal duplications comprising a region extending from NESP55 exon to AS exon 1 (but excluding the AS2 DMR). In cases with *STX16* deletions, NESP55 transcription is severely attenuated because of the lack of enhancer activity, as previously shown (18). For the patients with chromosomal duplications, it is conceivable that the duplicated NESP55 promoters compete for activation by the single shared *STX16* enhancer, as shown for other chromosomal loci (27, 28). Given that the STX16-ICR preferentially enhances the activity of the more closely located NESP55 promoter (i.e., centromeric of the duplicated promoters), the transcription from the second NESP55 promoter, which is closer to the AS2 DMR, would be attenuated. Consistent with our findings in hESC showing that AS2 methylation depends on STX16- and NESP-ICRs, these findings from the clinical samples suggest that the transcription from the NESP55 exon is required for AS2 methylation.

On the other hand, patients with defects affecting NESP55 transcription telomeric (downstream) of the AS2 DMR showed normal AS2 methylation levels. Our reporter assays using hESCs indicated that insertion of portions of a conserved retrotransposon blunts transcription. Since the retrotransposon insertion is associated with isolated, albeit variable, hypomethylation restricted to the A/B DMR on the maternal allele, it is plausible that the presence of the retrotransposon with its polyadenylation signals blunted NESP55 transcription after passing through the AS2 DMR, thereby preserving AS2 methylation while causing A/B hypomethylation. Consistent with this conclusion, patient-derived induced pluripotent stem cells showed lower expression levels of the NESP55 transcript in 1 of the 2 reported kindreds with retrotransposon insertion telomeric (downstream) of the AS2 DMR (12). Similarly, a chromosomal inversion involving A/B with a telomeric breakpoint between AS2 and A/B is likely to preserve NESP55 transcription passing through the AS2 DMR, whereas this transcript is unlikely to extend to the A/B DMR situated 1.8 million bp more telomeric (downstream) because of the inversion (11).

The presented clinical and epigenetic data collectively support our hypothesis that AS2 methylation depends on read-through NESP55 transcription controlled by the *STX16* enhancer, similar to the mechanism governing A/B methylation (18, 21). Based on these findings, we propose a mechanism-based categorization of PHP1B. While the loss of methylation at A/B is present in all patients with PHP1B, AS2 methylation status can help identify the location of genetic alterations that potentially blunt NESP55 transcription. By further distinguishing broad *GNAS* methylation defects, the current findings enable classificiation of *GNAS* methylation defects in PHP1B into categories 1–3 (Figure 6). Patients in category 1 show broad *GNAS* methylation changes through unknown mechanisms. Although the loss of methylation at the AS2 DMR suggests disrupted NESP55 transcription, defects underlying hypomethylation at the AS (AS1) and the XL DMRs remain to be elucidated. From a clinical perspective, sporadic PHP1B cases with undetermined genetic causes constitute the majority of patients in this category. It is plausible that some patients with PHP1B in this category have an inherited genetic defect, since NESP55-AS exons 3/4 deletion also leads to similar broad methylation defects (10). These heritable cases would be detected by copy number loss in MLPA unless only AS exons 3 and 4 are deleted (17). PHP1B cases in the remaining categories should be regarded as hereditary, and the genetic examination focusing on the specific chromosomal region should be performed based on the methylation patterns. Category 2 cases have genetic alterations disrupting NESP55 transcription centromeric (upstream) of the AS2 DMR. *STX16* deletions, leading to the loss of an enhancer for NESP55 transcription, are the most common cause in this category (9). Other less frequent causes include duplications spanning the NESP55 promoter (13), in which the telomeric (downstream) copy of the duplicated NESP55 promoters that normally dictates AS2 methylation is unlikely to be sufficiently active (as explained above). Deletions restricted to NESP55 exon and/or its promoter, which reportedly show a similar pattern of *GNAS* methylation defect as *STX16* deletions (15, 22), may also belong to this category; however, such samples were not available for our current study. Category 3 reflects genetic alterations that disrupt NESP55 transcription between AS2 and A/B. Therefore, the region between AS2 and A/B should be carefully investigated to search for defects like insertions or inversions (11, 12, 14, 20). This categorization would enable a mechanism-based approach to search for an unknown genetic cause in patients with PHP1B among the various alterations that lead to *GNAS* imprinting defects.

There are some unanswered questions in this study. First, the functional role of AS2 remains to be elucidated. Although it might operate as the XL promoter based on its chromosomal location, as discussed earlier, experimental evidence is lacking that would support this conclusion. Second, the mechanistic basis for category 1, namely broad methylation defects, remains unclear, except for the UPDpat involving chromosome 20q (4, 5). Murine studies suggested that methylation at Nesp55 DMR depends on the AS transcript and that the methylation of the AS1 and the XL DMRs depends on Nesp55 transcription during oogenesis (21, 29, 30). However, whether this applies to humans is unclear because the genomic imprinting mechanisms show considerable differences between rodents and humans (18, 31–33). Third, although we were able to analyze a wide variety of patients with PHP1B, only a few samples were available for patients with rare genetic defects — e.g., chromosomal inversions and duplications. The robustness of the categorization, thus, needs to be validated in more PHP1B cases.

In conclusion, based on the mechanistic findings of AS2 methylation, we propose the mechanism-based categorization of PHP1B. Using the 320 probe in a commercially available MS-MLPA assay, this categorization is widely applicable to guide the molecular diagnosis of PHP1B.

## Methods

### Sex as a biological variable.

The sex of the patients and unaffected controls was not considered as a biological variable in this study.

### Patients.

Patient characteristics are summarized in Supplemental Table 1. Thirty-one patients with PHP1B, including previously described cases (9, 11, 13, 14, 20, 34–36), and 21 unaffected controls were included. Patients were clinically diagnosed as PHP1B based on elevated PTH levels with or without hypocalcemia, hyperphosphatemia, and normal renal function. Molecular diagnosis of PHP1B was confirmed by hypomethylation at the *GNAS* A/B: TSS-DMR by MS-MLPA, as described below. We regarded PHP1B cases as sporadic and genetically undefined when patients had normal copy numbers in the *STX16* and *GNAS* regions as determined by MS-MLPA and when there was no family history of PHP (Supplemental Table 1); as outlined in our previous report (and observed in unpublished findings), siblings and offspring of family members of such sporadic PHP1B cases showed no *GNAS* methylation changes or laboratory abnormalities (37). In genetically undefined cases, paternal uniparental isodisomy involving a large portion of chromosome 20 was unlikely based on microsatellite analysis (Supplemental Table 1), as we have previously described (38). Unaffected controls, who showed normal *GNAS* methylation patterns, included unrelated spouses of mothers of AD-PHP1B patients, unaffected parents of patients with sporadic PHP1B, or unaffected children or siblings of sporadic or AD-PHP1B patients.

### DNA methylation analyses.

Genomic DNA was extracted from the leukocyte or buccal mucosa of the patients or normal controls using proteinase K digestion followed by phenol-chloroform extraction or DNeasy blood and tissue kit (QIAGEN). MS-MLPA was performed using the SALSA MS-MLPAProbemix ME031 GNAS (MRC Holland) according to the manufacturer’s protocol. Fragment analysis was conducted in the MGH DNA Core using ABI3730xl Genetic Analyzer, and the data were analyzed using the GeneMapper v6.0 software. MSRE-qPCR was performed as previously described, with minor modifications (18, 39). Briefly, 20 ng of genomic DNA was digested by 5U of HpaII (New England Biolabs) at 37°C for 2 hours under the presence of 1× rCutsmart buffer (New England Biolabs). qPCR of HpaII-digested or undigested control (mock) samples was performed using KOD SYBR (TOYOBO) on Quantstudio3.0 (Thermo Fisher Scientific). A calibration line was generated using the Ct values of serially diluted mock samples, and the relative amount of the digested sample was calculated.

### PHP1B model hESCs.

HESCs (HUES62 cells) were obtained from Harvard Stem Cell Research Institute and maintained on mTeSR1 plus (STEMCELL Technologies). PHP1B model hESCs were described in detail in our previous work (18). Briefly, for generating deletions of STX16-ICR or NESP-ICR, 2 gRNAs flanking the target region were introduced with Cas9 protein by nucleofection using 4D Nucleofector (Lonza). Following single-cell sorting using FACSAriaII (BD Biosciences), each clone was amplified and genotyped. The parental origin of the deleted allele was determined based on a heterozygous SNP within the deleted region, as we described (18).

### Luciferase assay.

Backbone plasmids were described previously (18). Reporter plasmids were constructed by inserting the PCR-amplified sequence derived from kindred #1, including the portion of retrotransposon before the firefly coding sequence. Firefly and Renilla plasmids were cotransfected to hESCs using lipofectamine 3000 (Thermo Fisher Scientific). Forty-eight hours after transfection, luciferase counts were measured using a Dual-Glo luciferase assay kit (Promega) and ENVISION (PerkinElmer). Firefly counts were normalized by Renilla counts.

### Statistics.

Statistical analyses were performed using GraphPad Prism 9 software. Data are shown as mean ± SEM. One-way ANOVA with the Dunnett multiple comparison test was used for multiple-group comparisons. Pearson correlation analysis was performed to analyze the correlation between 2 methylation values. The one-sample *t* test, 2-tailed, was used to compare methylation levels between WT hESCs and multiple clones of genome-edited hESCs. *P* < 0.05 were considered statistically significant.

### Study approval.

Written informed consent was obtained from each patient and unaffected control; IRB protocol no: 2001P000648. All experiments were approved by the Institutional Biosafety Committee of Mass General Brigham (no. 2019B000050).

### Data availability.

All data presented in this manuscript are accessible in the Supporting Data Values file or by request to the corresponding author.

## Author contributions

YI, MR, HJ, and MB conceived the study, designed the experiments, and interpreted data; YI drafted and edited the manuscript with input from all the authors.

## Supplementary Material

Supplemental data

Supporting data values

## Version 1 01/30/2024

In-Press Preview

## Version 2 03/08/2024

Electronic publication

## References

[1] Bastepe M (2017). Heterotrimeric G proteins in the control of parathyroid hormone actions. J Mol Endocrinol.

[2] Boyce AM, Collins MT (2020). Fibrous dysplasia/McCune-Albright syndrome: a rare, mosaic disease of Gαs activation. Endocr Rev.

[3] O’Hayre M (2013). The emerging mutational landscape of G proteins and G-protein-coupled receptors in cancer. Nat Rev Cancer.

[4] Mantovani G (2018). Diagnosis and management of pseudohypoparathyroidism and related disorders: first international Consensus Statement. Nat Rev Endocrinol.

[5] Jüppner H (2021). Molecular definition of pseudohypoparathyroidism variants. J Clin Endocrinol Metab.

[6] Albright F (1942). Pseudohypoparathyroidism–an example of ’Seabright-Bantam syndrome’ report of three cases. Endocrinology.

[7] Mantovani G (2002). The gsalpha gene: predominant maternal origin of transcription in human thyroid gland and gonads. J Clin Endocrinol Metab.

[8] Turan S (2014). Postnatal establishment of allelic Gαs silencing as a plausible explanation for delayed onset of parathyroid hormone resistance owing to heterozygous Gαs disruption. J Bone Miner Res.

[9] Bastepe M (2003). Autosomal dominant pseudohypoparathyroidism type Ib is associated with a heterozygous microdeletion that likely disrupts a putative imprinting control element of GNAS. J Clin Invest.

[10] Bastepe M (2005). Deletion of the NESP55 differentially methylated region causes loss of maternal GNAS imprints and pseudohypoparathyroidism type Ib. Nat Genet.

[11] Grigelioniene G (2017). A large inversion involving GNAS Exon A/B and all exons encoding Gsα is associated with autosomal dominant pseudohypoparathyroidism type Ib (PHP1B). J Bone Miner Res.

[12] Kawashima S (2022). Familial pseudohypoparathyroidism type IB associated with an SVA retrotransposon insertion in the GNAS locus. J Bone Miner Res.

[13] Reyes M (2021). A novel GNAS duplication associated with loss-of-methylation restricted to Exon A/B causes pseudohypoparathyroidism type Ib (PHP1B). J Bone Miner Res.

[14] Miller DE (2022). Targeted long-read sequencing identifies a retrotransposon insertion as a cause of altered GNAS Exon A/B methylation in a family with autosomal dominant pseudohypoparathyroidism type 1b (PHP1B). J Bone Miner Res.

[15] Danzig J (2021). High-throughput molecular analysis of pseudohypoparathyroidism 1b patients reveals novel genetic and epigenetic defects. J Clin Endocrinol Metab.

[16] Takatani R (2015). Similar frequency of paternal uniparental disomy involving chromosome 20q (patUPD20q) in Japanese and Caucasian patients affected by sporadic pseudohypoparathyroidism type Ib (sporPHP1B). Bone.

[17] Chillambhi S (2010). Deletion of the noncoding GNAS antisense transcript causes pseudohypoparathyroidism type Ib and biparental defects of GNAS methylation in cis. J Clin Endocrinol Metab.

[18] Iwasaki Y (2023). The long-range interaction between two GNAS imprinting control regions delineates pseudohypoparathyroidism type 1B pathogenesis. J Clin Invest.

[19] Rochtus A (2016). Genome-wide DNA methylation analysis of pseudohypoparathyroidism patients with GNAS imprinting defects. Clin Epigenetics.

[20] Hanna P (2021). A novel familial PHP1B variant with incomplete loss of methylation at GNAS-A/B and enhanced methylation at GNAS-AS2. J Clin Endocrinol Metab.

[21] Chotalia M (2009). Transcription is required for establishment of germline methylation marks at imprinted genes. Genes Dev.

[22] Richard N (2012). A new deletion ablating NESP55 causes loss of maternal imprint of A/B GNAS and autosomal dominant pseudohypoparathyroidism type Ib. J Clin Endocrinol Metab.

[23] Monk D (2019). Genomic imprinting disorders: lessons on how genome, epigenome and environment interact. Nat Rev Genet.

[24] Yang H (2022). Allele-specific H3K9me3 and DNA methylation co-marked CpG-rich regions serve as potential imprinting control regions in pre-implantation embryo. Nat Cell Biol.

[25] Yagi M (2019). De novo DNA methylation at imprinted loci during reprogramming into naive and primed pluripotency. Stem Cell Reports.

[26] Coombes C (2003). Epigenetic properties and identification of an imprint mark in the Nesp-Gnasxl domain of the mouse Gnas imprinted locus. Mol Cell Biol.

[27] Cho SW (2018). Promoter of lncRNA gene PVT1 is a tumor-suppressor DNA boundary element. Cell.

[28] Deng W (2014). Reactivation of developmentally silenced globin genes by forced chromatin looping. Cell.

[29] Williamson CM (2006). Identification of an imprinting control region affecting the expression of all transcripts in the Gnas cluster. Nat Genet.

[30] Fröhlich LF (2010). Targeted deletion of the Nesp55 DMR defines another Gnas imprinting control region and provides a mouse model of autosomal dominant PHP-Ib. Proc Natl Acad Sci U S A.

[31] Chu C (2021). Analysis of developmental imprinting dynamics in primates using SNP-free methods to identify imprinting defects in cloned placenta. Dev Cell.

[32] Cheong CY (2015). Germline and somatic imprinting in the nonhuman primate highlights species differences in oocyte methylation. Genome Res.

[33] Frohlich LF (2007). Lack of Gnas epigenetic changes and pseudohypoparathyroidism type Ib in mice with targeted disruption of syntaxin-16. Endocrinology.

[34] Kiuchi Z (2022). Progression of PTH resistance in autosomal dominant pseudohypoparathyroidism type Ib due to maternal STX16 deletions. J Clin Endocrinol Metab.

[35] Gruters-Kieslich A (2017). Early-onset obesity: unrecognized first evidence for GNAS mutations and methylation changes. J Clin Endocrinol Metab.

[36] Linglart A (2005). A novel STX16 deletion in autosomal dominant pseudohypoparathyroidism type Ib redefines the boundaries of a cis-acting imprinting control element of GNAS. Am J Hum Genet.

[37] Takatani R (2016). Analysis of multiple families with single individuals affected by pseudohypoparathyroidism type Ib (PHP1B) reveals only one novel maternally inherited GNAS deletion. J Bone Miner Res.

[38] Fernandez-Rebollo E (2011). Exclusion of the GNAS locus in PHP-Ib patients with broad GNAS methylation changes: evidence for an autosomal recessive form of PHP-Ib?. J Bone Miner Res.

[39] Keidai Y (2022). Sporadic pseudohypoparathyroidism type 1B in monozygotic twins: insights into the pathogenesis of methylation defects. J Clin Endocrinol Metab.

