# Supplementary material for: GNAS AS2 methylation status enables mechanism-based categorization of pseudohypoparathyroidism type 1B
Source: JCI Insight. 2024 Jan 30;9(5):e177190. doi: 10.1172/jci.insight.177190 (PMC10972583; doi:10.1172/jci.insight.177190)
Supplement: Supplemental data [file jciinsight-9-177190-s196.pdf]

Supplemental Table 1. PHP1B patients included in this study

Phenotype /genetic alterations	Patients	Family history	Microsatellite analysis	MLPA (copy number)		MS-MLPA (% methylation)						AS2 MSREqPCR (% methylation)	Age at diagnosis (yrs)	PTH (pg/ml)	Ca (mg/dl) ionized Ca (mmol/l)	P (mg/dl)	Ref
			UPD20	*STX16* deletion	*GNAS* deletion	NESP55 (average)	AS 256 probe	AS 166 probe	AS 320 probe	XL (average)	A/B (average)						
Sporadic PHP1B, complete methylation changes	300/II-1	-	Het	-	-	98.5	1.5	11.8	5.9	7.8	0.3		8.0	347	6.40	8.70	Unpublished
	301/II-1	-	Het	-	-	89.8	3.5	12.8	7.1	6.4	0.3		10.0	>500	6.60	8.22	Unpublished
	304/II-1	-	Het	-	-	98.8	1.8	8.0	5.6	10.6	0.9		12.0	584	5.40	10.00	Unpublished
	306/II-1	-	Het	-	-	94.3	2.9	11.4	6.2	2.8	0.3		12.0	490	5.80	6.70	Unpublished
	307/II-1	-	Het	-	-	86.3	9.7	17.1	7.5	6.5	3.4		13.0	449	7.70	5.90	Unpublished
	310/II-1	-	Het	-	-	104.5	3.4	12.4	10.8	4.3	0.6	0.6	9.0	376	7.50	8.80	Unpublished
	313/II-1	-	Het	-	-	101.8	4.1	11.3	14.9	4.3	2.4		N/A	N/A	N/A	N/A	Unpublished
	317/II-1	-	n.d.	-	-	97.7	2.1	10.4	7.8	21.0	0.3	0.0	21.0	284	0.67 (ion)	7.56	Unpublished
	325/II-1	-	Het	-	-	102.2	2.9	16.7	10.2	27.1	0.8		28.0	991	7.10	3.50	Unpublished
Sporadic PHP1B, incomplete methylation changes	318/II-1	-	n.d.	-	-	68.4	38.6	36.9	37.7	44.5	31.6	19.9	42.0	608	8.70		Unpublished
	321/II-1	-	Het	-	-	85.7	28.7	53.1	43.7	34.1	23.3		13.0	473	0.95 (ion)	7.20	Unpublished
Paternal UPD20	322/II-1	-	Hom	-	-	103.1	2.6	14.8	9.7	3.6	0.5		13.0	360	4.90	10.81	Unpublished
	303/II-1	-	Hom	-	-	90.3	0.0	14.7	6.6	1.5	0.0	2.0	14.0	337	5.40	7.80	Unpublished
	327/II-1	-	Hom	-	-	97.4	3.3	10.1	7.3	2.9	0.6		12.0	282	8.90	6.90	Unpublished
NESP55-AS exons 3/4 deletion	Y2/II-1	+	see ref	-	+	100.1	5.4	13.4	12.7	3.8	0.7	0.2					10
	C-II-1	+	see ref	-	+	99.2	2.0	9.7	7.8	3.1	0.3	0.7					
STX16 3kb deletion	297/II-1	-	n.d.	+	-	50.2	51.2	48.2	14.9	48.4	0.8		N/A	N/A	N/A	N/A	Unpublished
	173/II-2	+	n.d.	+	-	53.0	51.4	52.1	24.3	50.9	0.8						35
	173/II-2	+	n.d.	+	-	52.5	52.2	53.8	27.2	51.1	1.8						
	302/II-1	-	n.d.	+	-	44.5	48.7	47.1	20.4	51.1	0.0	0.5	49.0	370	6.69	2.69	Unpublished
	F-58	+	n.d.	+	-	49.5	50.2	49.9	21.8	52.2	0.7						34
	36/IV-6	+	n.d.	+	-	50.2	51.6	69.8	27.9	50.3	1.3		1.6	81.9	10.60		
STX16 4.4 kb deletion	W-12	+	Het	+	-	49.7	48.3	52.5	18.9	50.2	1.9	1.1					36
Duplication comprising the maternal NESP55-AS exon 1	249/II-1	+	see ref	-	-	36.6	48.0	47.3	18.1	52.1	2.4	0.8					13
Retrotransposon insertion	208/II-3	+	Het	-	-	53.1	52.8	56.6	54.0	52.6	18.1	44.0					14, 20
	208/III-3	+	Het	-	-	50.9	51.7	48.0	53.0	50.8	27.3	50.5					
	208/II-1	+	n.d.	-	-	52.6	50.8	49.2	54.2	53.1	1.9						
	208/II-2	+	Het	-	-	52.9	50.5	53.1	53.2	51.8	16.8						
	208/II-4	+	Het	-	-	51.3	56.4	59.3	51.5	47.6	10.9						
	208/III-1	+	Het	-	-	52.8	53.9	49.9	56.0	53.4	5.3						
Inversion with a centromeric breakpoint telomeric of XL	131/III-2	+	Het	-	-	50.7	48.9	50.2	47.0	49.1	0.6	20.7					11

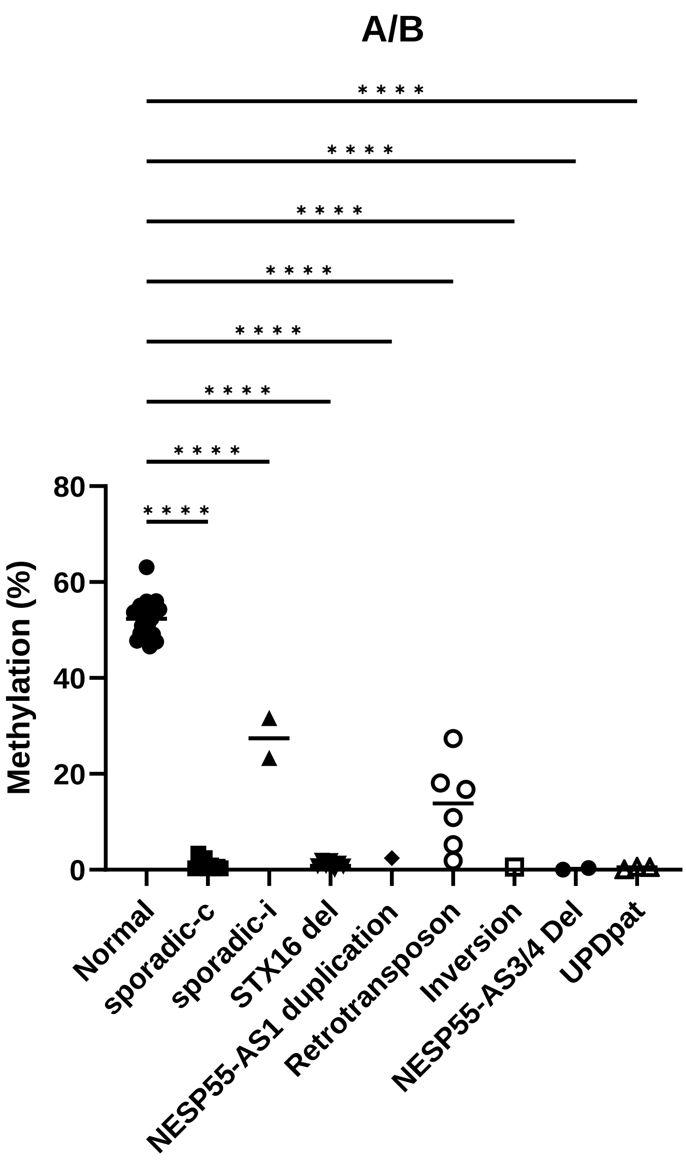

Supplemental Figure 1. Methylation levels at the A/B DMR in PHP1B patients and unaffected controls.

MS-MLPA results of PHP1B patients (n = 31 in total) and unaffected controls (n = 21). Methylation levels at the A/B DMR (the average values of three probes designed within the A/B DMR) are shown.

Sporadic-c, sporadic cases with complete methylation defects; Sporadic-i, sporadic cases with incomplete methylation defects; UPDpat, paternal uniparental disomy of chromosomal 20; NESP55-AS3/4 del, maternal deletion of NESP55-AS exons 3/4 region; STX16 del, maternal *STX16* deletion; NESP55-AS1 duplication, duplication comprising the maternal NESP55-AS exon 1 (excluding the region between the AS exon1 and the XL exon); Retrotransposon insertion, retrotransposon insertion telomeric (downstream) of the maternal XL exon; Inversion, maternal inversion involving A/B and all Gsα exons with a centromeric (upstream) breakpoint between XL and A/B. Intergroup comparisons were performed by one-way ANOVA with post hoc Dunnett multiple comparison test. **** $p < 0.0001$.

```
#1 2169  CTGGGAAGTGAGGAGCCCCTCTGCCCGGCCACGACCCCGTCTGGGAGGTGTGCCCAGCGG  2228
         |||||||||||||||||||||||||||||||| ||||||||||||||||||||||| | |
#2 317   CTGGGAAGTGAGGAGCCCCTCTGCCCGGCCACCACCCCGTCTGGGAGGTGTGCCCAACAG  376

   2229  CTCATTGGGGATGGGCCATGATGACAATGGCGGTTTTGTGGAATAGAAAGGCGGGAAGGG  2288
         ||||||| | | |||||| ||||||| |||||| ||||||||||||||||||||||| ||
   377   CTCATTGAGAACGGGCCAGGATGACAGTGGCGGCTTTGTGGAATAGAAAGGCGGGAAAGG  436

   2289  TGGGGAAAA-ATTGAGAAATCGGATGGTTGCGGGGTCTGTGTGGATAGAAGTAGACATGG  2347
         ||||||||| ||||||||||||||||||||| | |||||||| || ||||||||||||||
   437   TGGGGAAAAGATTGAGAAATCGGATGGTTGCCGTGTCTGTGTAGAAAGAAGTAGACATGG  496

   2348  GAGACTTTTCATTTTGTTCTGTACTAAGAAAAATTCTTCTGCCTTGGGATCCTGTTGATC  2407
         ||||||||||||||||||||| |||||||||||||| |||||||||||||||||||||||
   497   GAGACTTTTCATTTTGTTCTGCACTAAGAAAAATTCCTCTGCCTTGGGATCCTGTTGATC  556

   2408  TGTGACCTTATCCCCAACCCTGTGCTCTCTGAAACATGTGCTGTGTCCACTCAGGGTTAA  2467
         |||||||||| |||||| |||||||||||||||||||||||||||||||||||||||| |
   557   TGTGACCTTACCCCCAATCCTGTGCTCTCTGAAACATGTGCTGTGTCCACTCAGGGTTGA  616

   2468  ATGGATTAAGGGCGGTGCAAGATGTGCTTTGTTAAACAGATGCTTGAAGGCAGCATGCTC  2527
         ||||||||||||| |||||||||||||||||||  |||||||||||||||||||||||||
   617   ATGGATTAAGGGCAGTGCAAGATGTGCTTTGTTGGACAGATGCTTGAAGGCAGCATGCTC  676

   2528  GTTAAGAGTCATCACCACTCCCTAATCTTAAGTACCCAGGGACACAAACACTGCGGAAGG  2587
         ||||||||||||||||| |||||||||| |||||  ||||||||||||||||||||||||
   677   GTTAAGAGTCATCACCAATCCCTAATCTCAAGTAATCAGGGACACAAACACTGCGGAAGG  736

   2588  CCGCAGGGTCCTCTGCCTAGGAAAACCAGAGAGAGACCTTTGTTCACTTGTTTATCTGCT  2647
         |||| ||||||||||||||||||||||    |||||||||||||||||||||| ||||||
   737   CCGCGGGGTCCTCTGCCTAGGAAAACC----AGAGACCTTTGTTCACTTGTTTGTCTGCT  792

   2648  GACCTTCCCTCCACTATTGTCCTATGACCCTGCCAAATCCCCCTCTGCGAGAAACACCCA  2707
         |||||||||||||||||||||| ||||||||||||||||||||||||| |||||||||||
   793   GACCTTCCCTCCACTATTGTCCCATGACCCTGCCAAATCCCCCTCTGTGAGAAACACCCA  852

   2708  AGAATGATCAATaaaaataaaaaataaaaaataaaaaaaaaaaaaaaaaaaaaaaaaa  2764
         ||||| ||||||||||| || ||||  |||| |||||||||||||||| ||||||||
   853   AGAATTATCAATAAAAA-AATAAATTTAAAA-AAAAAAAAAAAAAAAAGAAAAAAAA  907
```

Supplemental Figure 2. Alignment of SVA transposon sequences identified in two unrelated kindreds.

Alignment result of a homologous region in SVA transposon sequences (described in Fig. 5B) is shown.
